# Etelcalcetide Versus Cinacalcet for Secondary Hyperparathyroidism in Hemodialysis Patients: A Multicentre Comparison with 24 Months of Follow-Up

**DOI:** 10.3390/jcm15176656

**Published:** 2026-08-28

**Authors:** Hakkı Öztürk, Çiğdem Ikhlef, Berrak Itır Aylı, Veysel Baran Tomar, Bartu Ediz, Ebru Gök Oğuz, Elif Arı Bakır, Galip Güz, Mehmet Deniz Aylı

**Affiliations:** 1 Afyonkarahisar Provincial Health Services Directorate, Afyonkarahisar 03200, Türkiye; 2 Division of Nephrology, Department of Internal Medicine, Ankara Etlik City Hospital, Ankara 06170, Türkiye; 3 Faculty of Life Sciences, University of Westminster, London W1B 2HW, UK; 4 Department of Nephrology, Faculty of Medicine, Gazi University, Ankara 06570, Türkiye; 5 Department of Nephrology, Kartal Dr Lütfü Kırdar City Hospital, Istanbul 34865, Türkiye

**Keywords:** secondary hyperparathyroidism, etelcalcetide, cinacalcet, calcimimetic, hemodialysis, CKD-MBD, propensity score matching, comparative effectiveness, real-world evidence

## Abstract

**Background/Objectives**: Secondary hyperparathyroidism (SHPT) is a prevalent complication of end-stage kidney disease. Although etelcalcetide has demonstrated short-term superiority over cinacalcet in randomized trials, real-world comparative data beyond 12 months remain scarce. This study compared the long-term effectiveness of etelcalcetide versus cinacalcet in hemodialysis patients over 24 months using propensity score matching. **Methods**: This multicenter retrospective study screened 457 hemodialysis patients across five centers in Türkiye. Propensity score matching on age, sex, hemodialysis vintage, and baseline intact parathyroid hormone (iPTH) yielded balanced cohorts of 21 versus 21 for 12-month and 14 versus 14 for 24-month analyses. The primary outcome was percentage change in iPTH from baseline (%ΔPTH). Secondary outcomes included responder rates, KDIGO target achievement, response trajectory classification, and depth-of-response analysis. **Results**: At 12 months, etelcalcetide achieved significantly greater iPTH reduction (%ΔPTH: −50.5 ± 25.6% vs. −29.7 ± 40.6%; *p* = 0.047), with effect sizes increasing from medium to medium–large at 24 months (Cohen’s d: −0.612 to −0.779). Sustained response (≥30% iPTH reduction at both timepoints) was significantly higher with etelcalcetide (78.6% vs. 28.6%; *p* = 0.021), as was deep response (≥75% reduction) at 24 months (57.1% vs. 14.3%; *p* = 0.046). At 24 months, etelcalcetide demonstrated significantly lower serum calcium (*p* = 0.015), calcium–phosphate product (*p* = 0.023), and superior phosphate target attainment (85.7% vs. 28.6%; *p* = 0.004). Inter-patient response variability was nearly three-fold lower with etelcalcetide (CV: 42% vs. 151%). **Conclusions**: Etelcalcetide provided significantly greater, more sustained, and more consistent iPTH suppression than cinacalcet over 24 months, with additional benefits in mineral metabolism control. These findings support etelcalcetide as a potent therapeutic option for SHPT in hemodialysis patients and warrant confirmation in larger prospective trials.

## 1. Introduction

Chronic kidney disease (CKD) is a condition characterized by a gradual deterioration in the structure and function of the kidneys over time [1]. Globally, the prevalence of CKD across all stages is estimated to be 14.2% [2]. Whereas in Türkiye, the CREDIT study in 2011 calculated the national prevalence of CKD as 15.7% and the recent Global Burden of Disease (GBD) estimated a prevalence of 17%; one of the highest CKD rates worldwide [2,3].

Patients who have reached the stage known as end-stage kidney disease and require renal replacement therapy (RRT) to survive constitute less than 10% of all CKD cases. According to the GBD study, as of 2023, approximately 4.6 million people are receiving RRT worldwide, whereas in Türkiye, according to 2024 records, there were 92.461 patients living with RRT, a number that is expected to rise in the upcoming decade [4,5,6].

Secondary hyperparathyroidism (SHPT), which is characterised by an increase in serum parathyroid hormone (iPTH) levels, is one of the major complications in CKD, affecting almost half of chronic kidney disease patients in varying degrees [7]. SHPT is a component of chronic kidney disease mineral and bone disorder (CKD-MBD), characterised by an increase in serum parathyroid hormone levels and fibroblast growth factor 23 (FGF-23) concentrations, in addition to a decrease in 1,25(OH)2 vitamin D concentrations and abnormalities in serum calcium (Ca) and phosphate (P) levels [1,7,8,9,10]. SHPT progresses with CKD, worsening and becoming more common in later stages while additionally increasing cardiovascular and fracture risk [7,8,9].

KDIGO guidelines recommend maintaining iPTH at two to nine times the upper normal limit, lowering elevated phosphate into the normal range, and avoiding calcium overload [10]. For patients who require iPTH-lowering therapy, the options include calcimimetics, vitamin D analogs, or a combination of them. In cases of severe hyperparathyroidism that are unresponsive to medical treatment, a surgical approach in parathyroidectomy should be considered [10,11].

In the medical treatment of SHPT among patients undergoing dialysis, vitamin D analogues and calcimimetics are the two key therapeutic agents. Calcitriol, the active form of vitamin D, and paricalcitol, a selective vitamin D receptor activator, help suppress iPTH production by enhancing calcium absorption and directly regulating parathyroid gland activity [12,13]. Calcimimetics such as cinacalcet and the newer intravenous agent etelcalcetide act by increasing the sensitivity of calcium-sensing receptors (CaSR) on the parathyroid glands, leading to a reduction in iPTH without significantly raising calcium or phosphate levels. Cinacalcet is an oral medication and is often preferred in outpatient settings; however, treatment adherence to cinacalcet is dependent on the patient, whereas etelcalcetide, given post-dialysis intravenously, improves adherence while also reducing the pill burden that is especially high in hemodialysis cohorts [13,14]. In a pivotal head-to-head randomized controlled trial, etelcalcetide demonstrated superiority over cinacalcet in achieving >30% iPTH reduction (68.2% vs. 57.7%, *p* = 0.004), establishing its potential as a more effective calcimimetic option [15]. A subsequent network meta-analysis confirmed a favourable efficacy profile for etelcalcetide compared with cinacalcet, although both agents carried a similar risk of hypocalcemia [14]. However, these findings were derived predominantly from short-term controlled trials of 6–12 months duration, and real-world comparative data extending to 24 months remain scarce, particularly from non-Western populations.

Despite the growing body of evidence from randomized trials, several important gaps remain. First, most studies have reported outcomes limited to 6–12 months, with very few extending follow-up beyond a year in routine clinical practice. Second, direct comparisons between etelcalcetide and cinacalcet in real-world settings are limited, with available data coming primarily from high-income countries. Third, there is a paucity of data on calcimimetic use in Turkish hemodialysis patients, despite Türkiye having one of the highest RRT prevalence rates in Europe [4,5]. Fourth, to our knowledge, no prior study has employed propensity score matching to account for the confounding by indication that is inherent in observational calcimimetic comparisons, whereby etelcalcetide is preferentially prescribed for patients with more severe or treatment-refractory disease. In Türkiye, cinacalcet has been the mainstay of calcimimetic therapy for over a decade, while etelcalcetide has only recently become available, creating a natural opportunity to compare these agents in a real-world setting.

This propensity-score-matched multicenter retrospective study aims to evaluate the comparative effectiveness of etelcalcetide versus cinacalcet in hemodialysis patients with SHPT across five centers in Türkiye, with follow-up extending to 24 months. By comparing iPTH trajectories, responder rates, sustained and deep response patterns, mineral metabolism endpoints, and inter-patient response variability between the two treatment groups, this study provides long-term, propensity-matched, real-world comparative effectiveness data for these agents in a non-Western cohort.

## 2. Materials and Methods

### 2.1. Study Design and Setting

This was a multicentre, retrospective, cohort study conducted across five centres in Türkiye between January 2022 and January 2026. The participating centres included three etelcalcetide-prescribing centres (Centre A, Centre B, and Centre D), whereas cinacalcet was prescribed in all 5 centers. This was because etelcalcetide is only available in hospital settings in Türkiye; therefore, satellite dialysis centres use only cinacalcet. The study was designed as a real-world comparative effectiveness analysis using propensity score matching to minimise confounding between treatment groups. The study was approved by the Ankara Etlik City Hospital Ethics Committee (Approval Number: AEŞH-BADEK-2024-344; AESH-BADEK2-2026-708). Written informed consent was waived due to the retrospective design and the use of de-identified clinical data.

### 2.2. Study Population

A total of 457 maintenance hemodialysis patients were screened across the five participating centers. Among these, 21 patients were receiving etelcalcetide and 38 patients were receiving cinacalcet for the treatment of secondary hyperparathyroidism (SHPT) for at least 12 months.

Adult (≥18 years) maintenance hemodialysis patients who had received etelcalcetide for at least 12 months with available iPTH, serum calcium, and phosphate measurements at baseline and 12 months were eligible for inclusion. All 21 identified etelcalcetide users across three centers met these criteria and were included: Center A (n = 6), Center B (n = 10), and Center D (n = 5). Of these, 14 patients completed 24-month follow-up. Of the remaining 7, 5 had only been on treatment for 12 months, 1 underwent transplantation and 1 patient was lost to follow-up. Etelcalcetide was administered as an intravenous bolus at the end of each hemodialysis session, with dose titration guided by iPTH response and serum calcium levels at the discretion of the treating nephrologist.

Among the 38 cinacalcet-treated patients, 30 patients met the full inclusion criteria: continuous cinacalcet use for 24 months, with available iPTH, serum calcium, and phosphate values at baseline, 12 months, and 24 months. The 21 patients in the study were chosen according to propensity scores.

### 2.3. Propensity Score Matching

Given the non-randomized design and the inherent potential for confounding by indication, etelcalcetide is typically reserved for patients with more severe or cinacalcet-refractory hyperparathyroidism; propensity score matching was employed to optimize comparability between the two treatment groups.

#### 2.3.1. Propensity Score Estimation

Propensity scores were estimated using a logistic regression model. Four baseline covariates were selected a priori based on their potential to confound the treatment–outcome relationship: age, sex, hemodialysis vintage (years), and baseline iPTH (pg/mL). Continuous covariates were standardized (zero mean, unit variance) prior to model fitting to ensure stable coefficient estimation. The resulting propensity score represented the predicted probability of receiving etelcalcetide conditional on these baseline characteristics.

#### 2.3.2. Matching Procedure

Two separate matching procedures were performed for the 12-month and 24-month analyses.

For the 12-month analysis, 1:1 nearest-neighbor matching without replacement was performed. Each of the 21 etelcalcetide patients was sequentially matched to the cinacalcet patient with the closest propensity score.

For the 24-month analysis, the 14 etelcalcetide patients with complete 24-month follow-up constituted the fixed group. From the 21 matched cinacalcet patients in the 12-month cohort, 14 were selected using the same 1:1 nearest-neighbor matching procedure applied to the propensity scores of the 24-month etelcalcetide subset.

#### 2.3.3. Balance Assessment

Covariate balance after matching was assessed using two complementary approaches: (1) statistical testing via Mann–Whitney U tests (continuous variables) and Fisher’s exact test (sex), with a prespecified adequacy threshold of *p* > 0.10 for all covariates; and (2) standardized mean differences (SMD), where |SMD| < 0.25 was considered acceptable balance given the small sample size, acknowledging that the conventional threshold of |SMD| < 0.10 is more appropriate for larger cohorts. Post-matching balance was additionally verified for covariates not included in the propensity model (serum calcium, phosphate, and calcium–phosphate product). The complete propensity score distributions, matched-pair assignments, and SMD values are reported in Table S1.

### 2.4. Data Collection

Demographic and clinical data were extracted from medical records at each center and included age, sex, dialysis vintage, comorbidities, medication history (phosphate binders, vitamin D analogs, oral calcium supplementation, dialysate calcium concentration), calcimimetic dosing, and adverse events. Laboratory parameters of interest were intact parathyroid hormone (iPTH; measured by electrochemiluminescence immunoassay), serum calcium (corrected for albumin where available), and serum phosphate.

### 2.5. Outcome Measures

The primary outcome was the percentage change in iPTH from baseline (%ΔPTH), assessed at 12 months (21 vs. 21) and 24 months (14 vs. 14). This was calculated as: %ΔPTH = [(iPTH*timepoint* − iPTH*baseline*)/iPTH*baseline*] × 100.

Secondary outcomes included: (1) absolute change in iPTH from baseline (pg/mL); (2) proportion of patients achieving ≥30% and ≥50% iPTH reduction; (3) proportion achieving the KDIGO-recommended iPTH target range (150–600 pg/mL); (4) within-group iPTH, calcium, and phosphate trajectories over time; (5) between-group differences in serum calcium, phosphate, and calcium–phosphate (Ca × P) product at each timepoint; and (6) adverse events, with specific attention to hypocalcemia requiring dose modification or interruption.

The following analyses were prespecified as exploratory and hypothesis-generating, and their results should be interpreted accordingly: (1) response trajectory classification (sustained, late, transient, or non-responder based on response status at both 12 and 24 months); (2) depth-of-response analysis (categorizing patients by magnitude of iPTH reduction: deep [≥75%], good [50–74%], moderate [30–49%], minimal [<30%], or no response/increase); (3) a composite mineral metabolism endpoint (simultaneous attainment of KDIGO targets for iPTH, calcium, and phosphate at 24 months); (4) inter-patient response variability assessed by the coefficient of variation (CV) of %ΔPTH; and (5) subgroup analyses by baseline iPTH severity.

### 2.6. Statistical Analysis

Continuous variables were expressed as mean ± standard deviation (SD) and median with interquartile range (IQR). Categorical variables were expressed as frequencies and percentages.

Baseline characteristics were compared using the Mann–Whitney U test for continuous variables and Fisher’s exact test for categorical variables. Within-group changes across time points (baseline, 12, 24 months) were assessed using the Friedman test. When the Friedman test was significant, post-hoc pairwise Wilcoxon signed-rank tests were performed with Bonferroni correction for three comparisons (adjusted significance threshold: *p* < 0.017). Between-group comparisons used the Mann–Whitney U test due to non-normal iPTH distributions; 95% CIs for mean differences were computed using Welch’s t-method and may not align precisely with the rank-based *p*-values. Responder rates were compared using Fisher’s exact test. Odds ratios (OR) with 95% confidence intervals (CI) were calculated using the Wald log-OR method, with Haldane’s correction applied when any cell contained zero. The number needed to treat (NNT) was calculated as 1/ARD for endpoints favouring etelcalcetide. Effect sizes were quantified using Cohen’s d (thresholds: 0.2 small, 0.5 medium, 0.8 large). A two-sided *p* < 0.05 was considered statistically significant. No adjustment for multiplicity was applied to the between-group secondary and exploratory comparisons. These analyses are considered hypothesis-generating, and their *p*-values should be interpreted as descriptive rather than confirmatory. All analyses were performed using Python 3.10 with SciPy 1.11, scikit-learn 1.3, and pandas 2.0 on Google Colab.

### 2.7. Sample Size Considerations

The sample size was determined by the available patient pool and was not based on a formal a priori power calculation. Post-hoc power analysis for the primary outcome (%ΔPTH at 12 months, n = 21 per group) indicated 58% power to detect the observed effect size (Cohen’s d = 0.612) at two-sided α = 0.05. For the 24-month analysis (n = 14 per group), the observed d = 0.779 yielded 55% power. The study was designed as a hypothesis-generating real-world comparison. Effect estimates and their confidence intervals are reported as the primary basis for interpreting clinical significance, rather than reliance on *p*-value thresholds alone.

## 3. Results

### 3.1. Study Population and Baseline Characteristics

A total of 42 hemodialysis patients with secondary hyperparathyroidism were included from five centers across Türkiye. The etelcalcetide group comprised 21 patients (Center A: 6, Center B: 10, Center D: 5), while the cinacalcet group comprised 21 patients selected using a propensity-based balancing algorithm that minimized differences in age, sex, hemodialysis vintage, and baseline iPTH across groups.

Baseline characteristics are presented in Table 1. The two groups were well balanced across all measured baseline variables, with no statistically significant differences between groups in either the 12- or 24-month cohort.

### 3.2. Twelve-Month Outcomes

#### 3.2.1. iPTH Outcomes at 12 Months

Both calcimimetic agents produced significant reductions in iPTH from baseline to 12 months, though the magnitude of reduction differed substantially between groups. In the etelcalcetide group, mean iPTH decreased from 1831.5 ± 1117.9 to 935.4 ± 761.9 pg/mL, representing a mean absolute reduction of 896.1 pg/mL (Wilcoxon signed-rank *p* < 0.001). In the cinacalcet group, mean iPTH decreased from 1377.0 ± 811.9 to 910.7 ± 786.9 pg/mL, corresponding to a mean absolute reduction of 466.2 pg/mL (*p* = 0.003). Despite the approximately two-fold greater absolute reduction with etelcalcetide, the absolute iPTH levels at 12 months did not differ significantly between the groups (Table 2), reflecting the higher baseline iPTH in the etelcalcetide cohort.

**Figure 1:**
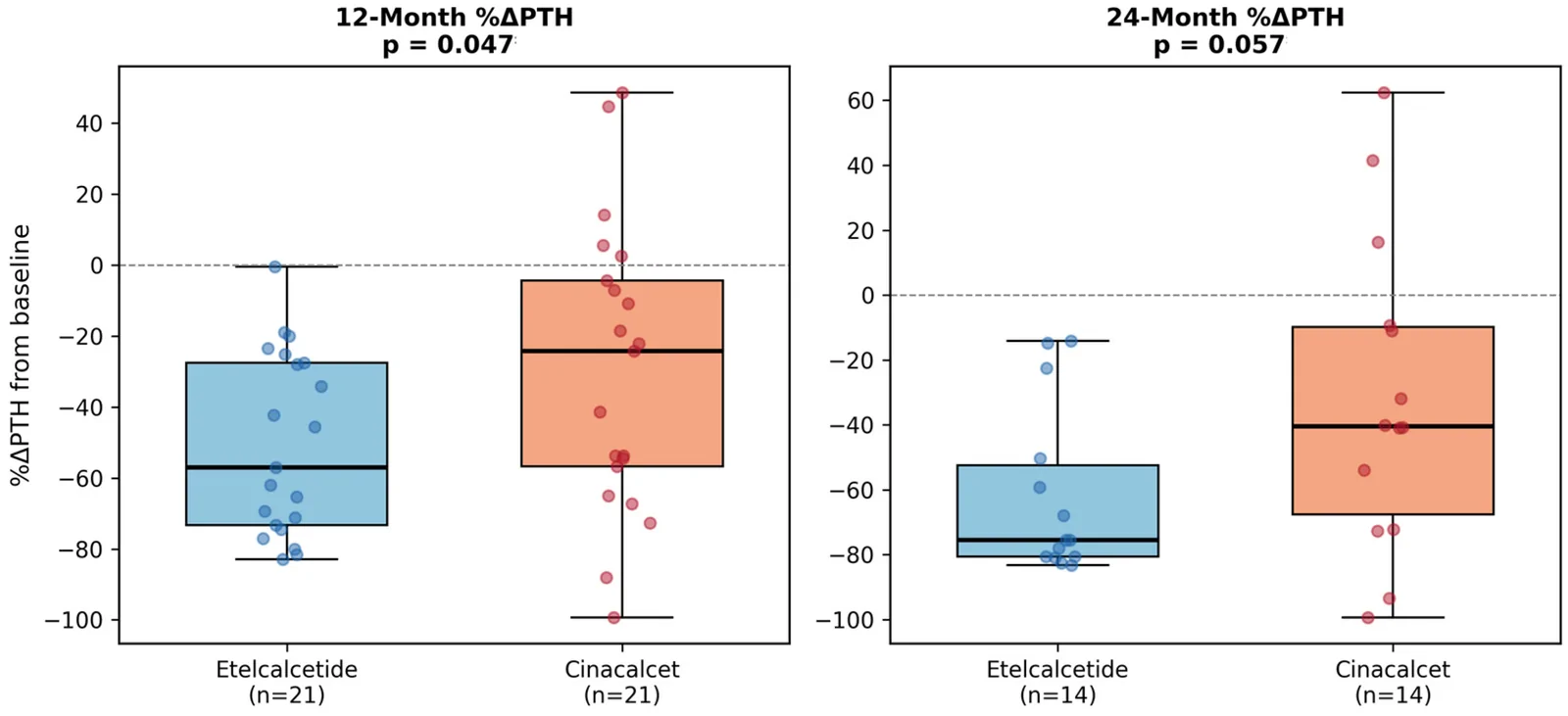
Box-and-strip plot of percentage change in iPTH (%ΔPTH) from baseline at 12 months (n = 21 per group) and 24 months (n = 14 per group).

The primary efficacy endpoint, percentage change in iPTH from baseline (%ΔPTH), demonstrated a significantly greater reduction with etelcalcetide than cinacalcet at 12 months (−50.5 ± 25.6% vs. −29.7 ± 40.6%; Mann–Whitney U test, *p* = 0.047; Table 3, Figure 1). This between-group difference was even more pronounced when assessed by absolute iPTH change (−896.1 ± 721.9 vs. −466.2 ± 757.4 pg/mL; *p* = 0.007), yielding a medium effect size (Cohen’s d = −0.581). The percentage-based comparison yielded a Cohen’s d of −0.612, also indicating a medium effect (Table S2). At 24 months (n = 14 per group), the between-group difference in %ΔPTH widened further (−61.9 ± 26.0% vs. −31.8 ± 47.9%; *p* = 0.057; Cohen’s d = −0.779), as detailed in Section 3.3.2 (Figure 1, right panel).

#### 3.2.2. Calcium, Phosphate, and Ca × P Product at 12 Months

Serum calcium remained stable in both groups at 12 months, with no significant within-group changes (etelcalcetide: Δ = −0.01 mg/dL, *p* = 0.422; cinacalcet: Δ = −0.23 mg/dL, *p* = 0.322) and no significant between-group difference (%ΔCa: +0.1 ± 11.0% vs. −2.4 ± 11.0%; *p* = 0.589). Serum phosphate declined in both groups, reaching significance only in the cinacalcet group (Δ = −0.63 mg/dL, *p* = 0.016; etelcalcetide: Δ = −0.43 mg/dL, *p* = 0.175), though the between-group comparison of percentage change was not significant (%ΔP: −4.9 ± 23.5% vs. −7.1 ± 27.3%; *p* = 0.450). The calcium–phosphate product did not differ significantly between groups at 12 months (48.9 ± 10.4 vs. 45.8 ± 11.6; *p* = 0.352).

#### 3.2.3. Responder Rates at 12 Months

Clinically defined responder rates, defined as the proportion of patients achieving prespecified thresholds of iPTH reduction (≥30% or ≥50%) or reaching the KDIGO target range (150–600 pg/mL), favored etelcalcetide at 12 months, though no comparison reached statistical significance in this sample (Table 2). A ≥30% reduction in iPTH was achieved by 14 of 21 patients (66.7%) in the etelcalcetide group versus 10 of 21 (47.6%) in the cinacalcet group (*p* = 0.350), yielding a number needed to treat (NNT) of 5.2. A ≥50% reduction was observed in 11 of 21 (52.4%) etelcalcetide-treated patients versus 9 of 21 (42.9%) cinacalcet-treated patients (*p* = 0.758; NNT = 10.5). Attainment of the KDIGO-recommended iPTH target (150–600 pg/mL) was achieved by 52.4% and 33.3% of patients, respectively (*p* = 0.350; odds ratio [OR] = 2.20; 95% CI: 0.63–7.66).

### 3.3. Twenty-Four-Month Outcomes

#### 3.3.1. iPTH Outcomes at 24 Months

**Figure 2:**
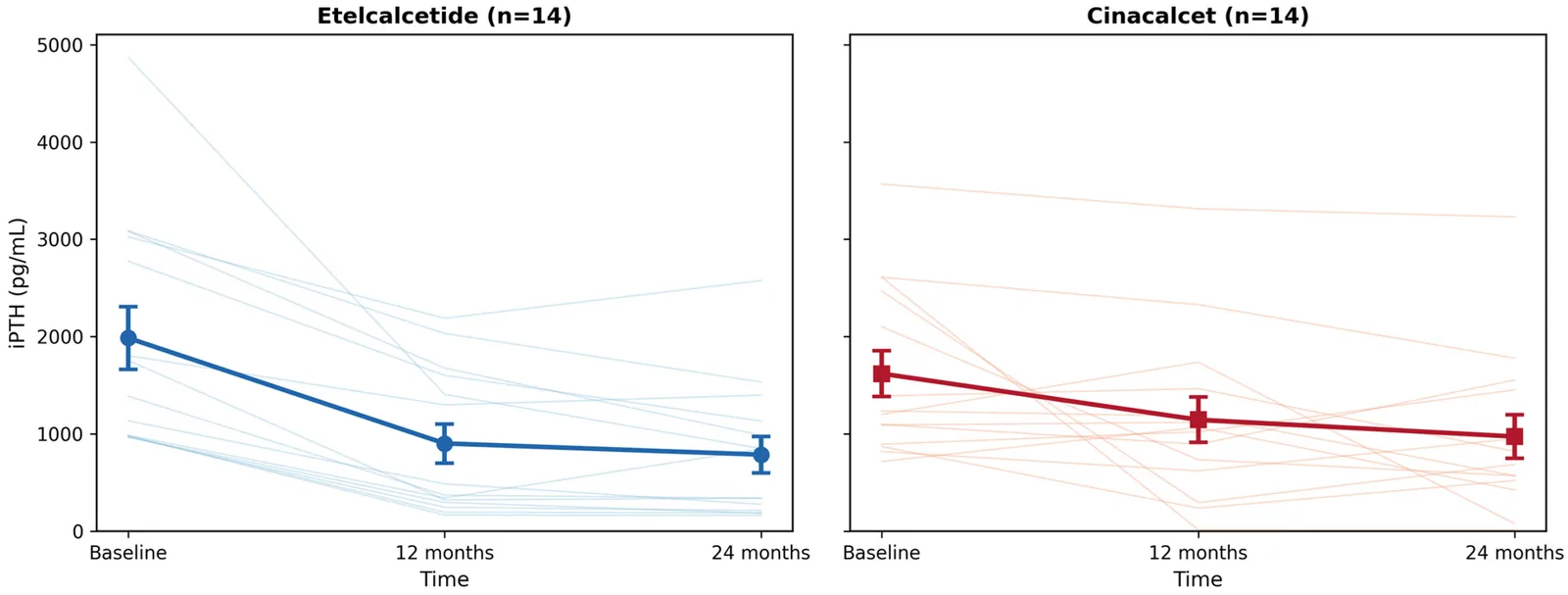
Individual and mean (±SEM) iPTH trajectories over 24 months. Translucent lines depict individual patients; bold lines with error bars denote group means.

In the 24-month matched subset (n = 14 per group), the Friedman test revealed a highly significant time effect on iPTH in the etelcalcetide group (χ^2^ = 22.29, *p* < 0.001) but only a borderline non-significant trend in the cinacalcet group (χ^2^ = 5.14, *p* = 0.076; Figure 2). Post-hoc pairwise comparisons with Bonferroni correction showed that etelcalcetide produced significant iPTH reductions at both 0 → 12 months (adjusted *p* < 0.001) and 0 → 24 months (adjusted *p* < 0.001), while the 12 → 24-month interval showed continued decline that did not independently reach significance (adjusted *p* = 0.518). In the cinacalcet group, only the 0 → 24-month comparison reached significance (adjusted *p* = 0.050), and the 12 → 24-month comparison was non-significant (adjusted *p* = 1.000), indicating a plateau in the iPTH-lowering response beyond the first year.

At 24 months, the between-group comparison of %ΔPTH showed a numerically larger reduction with etelcalcetide (−61.9 ± 26.0% vs. −31.8 ± 47.9%), approaching but not reaching significance (*p* = 0.057). The absolute iPTH change followed the same pattern (−1197.9 ± 971.3 vs. −645.9 ± 875.9 pg/mL; *p* = 0.085).

#### 3.3.2. Calcium, Phosphate, and Ca × P Product at 24 Months

**Figure 3:**
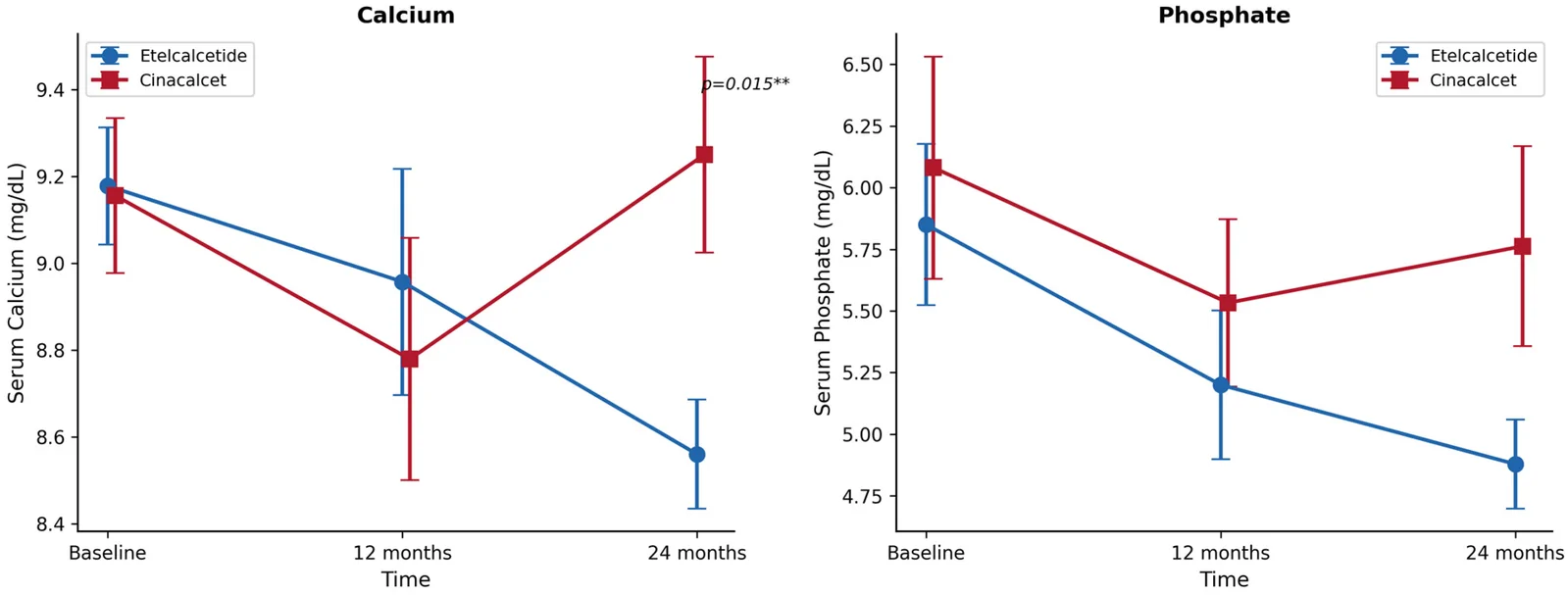
Mean (±SEM) calcium and phosphate trajectories over 24 months in the matched subset (n = 14 per group). Asterisks denote significant between-group differences at the indicated timepoint.

Serum calcium at 24 months was significantly lower in the etelcalcetide group (8.56 ± 0.47 vs. 9.25 ± 0.84 mg/dL; *p* = 0.015), with a corresponding significant between-group difference in percentage change from baseline (%ΔCa: −6.4 ± 8.3% vs. +1.6 ± 12.2%; *p* = 0.046; Figure 3). This calcium-lowering effect with etelcalcetide was consistent with the Friedman test, which demonstrated a significant time effect on calcium in the etelcalcetide group (χ^2^ = 9.51, *p* = 0.009) but not in the cinacalcet group (χ^2^ = 1.56, *p* = 0.458). Post-hoc analysis confirmed a significant baseline-to-24-month calcium decline with etelcalcetide (adjusted *p* = 0.039).

Serum phosphate showed a borderline between-group difference at 24 months (4.88 ± 0.68 vs. 5.76 ± 1.52 mg/dL; *p* = 0.069), with the Friedman test reaching significance only in the etelcalcetide group (χ^2^ = 6.65, *p* = 0.036 vs. χ^2^ = 3.57, *p* = 0.168). Post-hoc analysis confirmed a significant 0 to 24-month phosphate reduction with etelcalcetide (adjusted *p* = 0.027) but not with cinacalcet (adjusted *p* = 1.000). Percentage change in phosphate did not differ significantly between groups (%ΔP: −13.8 ± 18.3% vs. +0.2 ± 31.4%; *p* = 0.206).

The calcium–phosphate product at 24 months was significantly lower in the etelcalcetide group (41.8 ± 6.8 vs. 53.2 ± 14.6; *p* = 0.023), representing a clinically meaningful 22% relative difference that may have cardiovascular implications.

#### 3.3.3. Responder Rates at 24 Months

Responder rates at 24 months consistently favored etelcalcetide and showed a stronger between-group separation than at 12 months (Table 3). A ≥50% iPTH reduction was achieved by 11 of 14 patients (78.6%) in the etelcalcetide group compared with 5 of 14 (35.7%) in the cinacalcet group (*p* = 0.054; OR = 6.60, 95% CI: 1.23–35.44), with a NNT of 2.3; indicating that for every 2.3 patients treated with etelcalcetide instead of cinacalcet, one additional patient would achieve a clinically meaningful ≥50% iPTH reduction. A ≥30% iPTH reduction was observed in 78.6% vs. 64.3% (*p* = 0.678; NNT = 7.0), and KDIGO target attainment was 50.0% vs. 28.6% (*p* = 0.440; OR = 2.50, 95% CI: 0.52–11.93).

### 3.4. Exploratory Analyses

The following exploratory analyses were prespecified and are presented as hypothesis-generating; no adjustment for multiplicity was applied.

#### 3.4.1. Subgroup Analysis by Baseline iPTH Severity

In a post-hoc subgroup analysis stratified by the overall median baseline iPTH (1168 pg/mL), the advantage of etelcalcetide was numerically more pronounced in the lower-iPTH subgroup (%ΔPTH: −55.3% vs. −28.8%; *p* = 0.051) than in the higher-iPTH subgroup (−46.8% vs. −30.9%; *p* = 0.414), though the interaction should be interpreted with caution given the small subgroup sizes.

#### 3.4.2. Temporal Patterns of Response

In the etelcalcetide group, iPTH continued to decline between 12 and 24 months, although this incremental change did not reach significance on post-hoc testing (adjusted *p* = 0.518). In contrast, the cinacalcet group exhibited a plateau: iPTH declined modestly from baseline to 12 months and showed minimal further change between 12 and 24 months (adjusted *p* = 1.000). The Friedman test results further highlighted this pattern: all three laboratory parameters (iPTH, Ca, P) showed significant longitudinal trends in the etelcalcetide group (*p* < 0.001, *p* = 0.009, and *p* = 0.036, respectively), whereas none reached significance in the cinacalcet group.

#### 3.4.3. Composite Mineral Metabolism Endpoint

A composite endpoint was defined as simultaneous attainment of all three KDIGO mineral targets at 24 months: iPTH 150–600 pg/mL, serum calcium 8.4–10.2 mg/dL, and serum phosphate 3.5–5.5 mg/dL. This composite was achieved by 7 of 14 etelcalcetide patients (50.0%) compared with 2 of 14 cinacalcet patients (14.3%; *p* = 0.103; OR = 6.00, 95% CI: 0.97–37.30; Table S3).

#### 3.4.4. Consistency of Response

The coefficient of variation (CV) of %ΔPTH was used to assess the consistency of the treatment response across patients. Etelcalcetide demonstrated substantially lower inter-patient variability than cinacalcet at both timepoints. At 12 months (21 vs. 21), the CV was 50.6% for etelcalcetide versus 136.7% for cinacalcet; at 24 months (14 vs. 14), the CV was 42.1% versus 150.6%, respectively.

#### 3.4.5. Depth of Response

Deep response (≥75% iPTH reduction) at 24 months was achieved by 8 of 14 etelcalcetide patients (57.1%) versus 2 of 14 cinacalcet patients (14.3%; *p* = 0.046).

## 4. Discussion

### 4.1. Summary of Key Findings

To our knowledge, this multicentre retrospective study provides the long-term real-world comparative effectiveness data to date for etelcalcetide versus cinacalcet in the treatment of SHPT in hemodialysis patients. The principal findings can be summarised as follows. First, etelcalcetide produced a significantly greater percentage reduction in iPTH at 12 months (−50.5% vs. −29.7%; *p* = 0.047) and a clinically meaningful, near-significant advantage at 24 months (−61.9% vs. −31.8%; *p* = 0.057) compared with cinacalcet. Second, the rate of sustained response (≥30% iPTH reduction maintained at both 12 and 24 months) was significantly higher with etelcalcetide (78.6% vs. 28.6%; *p* = 0.021), representing the biggest between-group difference in this study. Third, etelcalcetide demonstrated a significantly higher deep response rate (≥75% iPTH reduction) at 24 months (57.1% vs. 14.3%; *p* = 0.046) and a substantially lower inter-patient response variability (CV: 42% vs. 151%). Fourth, etelcalcetide achieved superior mineral metabolism control, with significantly lower serum calcium (*p* = 0.015), calcium–phosphate product (*p* = 0.023), and phosphate target attainment (85.7% vs. 28.6%; *p* = 0.004) at 24 months. These findings extend the existing evidence base from randomised controlled trials [15,16] and short-term real-world studies [17,18] by demonstrating that the therapeutic advantage of etelcalcetide may be maintained or even amplified over a 24-month follow-up period.

### 4.2. Comparison with Existing Literature

The pivotal head-to-head randomized trial by Block et al. demonstrated the superiority of etelcalcetide over cinacalcet in achieving a ≥30% iPTH reduction at 26 weeks [15]. Our 12-month findings are broadly consistent with this direction of effect, with a ≥30% responder rate of 66.7% for etelcalcetide versus 47.6% for cinacalcet. The somewhat lower responder rate in our cinacalcet group may reflect the longer observation period, real-world adherence variability, and higher baseline disease severity compared with the trial population. Notably, the magnitude of iPTH reduction with etelcalcetide in our study (%ΔPTH: −50.5% at 12 months, −61.9% at 24 months) is comparable to the reduction rates reported in the placebo-controlled trials of etelcalcetide, suggesting that the efficacy observed in controlled settings is reproducible in routine clinical practice [16].

The one-year safety and efficacy data reported by Bushinsky et al. demonstrated sustained iPTH suppression with etelcalcetide over 52 weeks, with a median iPTH reduction of approximately 57% [17]. Our 12-month results are consistent with these findings. However, our study adds to the evidence by providing 24-month data, suggesting two important temporal patterns not captured in shorter studies: the continued decline in iPTH with etelcalcetide beyond 12 months and the concurrent plateau phenomenon in the cinacalcet group. The Friedman test confirmed this divergence: all three mineral parameters (iPTH, Ca, P) showed significant longitudinal trends in the etelcalcetide group (*p* < 0.001, *p* = 0.009, *p* = 0.036) but none reached significance in the cinacalcet group (*p* = 0.076, *p* = 0.458, *p* = 0.168).

The real-world Argentine study by Wojtowicz et al. also reported favorable outcomes with etelcalcetide in a routine clinical setting, with significant iPTH reductions and acceptable tolerability [18]. Our study adds to this growing body of real-world evidence from non-Western populations, extending the geographical and ethnic representativeness of etelcalcetide effectiveness data. The recent systematic review and meta-analysis by Nakai et al. further supports the calcium- and phosphate-lowering advantages of calcimimetics, though it did not specifically compare etelcalcetide with cinacalcet in long-term real-world settings [19]. Our composite mineral metabolism endpoint analysis, showing that 50.0% of etelcalcetide patients simultaneously achieved all three KDIGO mineral targets versus 14.3% with cinacalcet (OR = 6.00), provides a clinically relevant perspective not previously reported in this comparison.

### 4.3. Temporal Response Dynamics and Sustained Efficacy

One of the most clinically relevant findings of this study was the divergence in response trajectories between the two agents over time. In the etelcalcetide group, iPTH continued to decline between 12 and 24 months, with effect sizes increasing from Cohen’s d = −0.612 at 12 months to d = −0.779 at 24 months. The NNT for ≥50% iPTH reduction improved from 10.5 to 2.3, indicating that the clinical return per patient treated increases substantially with longer treatment duration. In contrast, the cinacalcet group exhibited a plateau phenomenon, with minimal further iPTH suppression beyond 12 months (post-hoc Wilcoxon 12 → 24 months: adjusted *p* = 1.000). This pattern is consistent with the pharmacological profile of oral cinacalcet, which is subject to variable gastrointestinal absorption, hepatic first-pass metabolism, and patient-dependent adherence [13,20]. Etelcalcetide, administered intravenously during each dialysis session, bypasses these sources of variability, ensuring consistent drug delivery and potentially allowing for continued receptor-mediated iPTH suppression over time.

The response trajectory classification revealed that 78.6% of etelcalcetide patients were sustained responders (meeting the ≥30% threshold at both timepoints) versus only 28.6% of cinacalcet patients (*p* = 0.021). Furthermore, the cinacalcet group exhibited a heterogeneous pattern, with 35.7% of patients classified as late responders, achieving ≥30% reduction only at 24 months after failing to do so at 12 months. This delayed response pattern suggests that cinacalcet’s effect may be less predictable, possibly reflecting dose titration challenges or fluctuating adherence patterns that gradually improve with patient education and clinical follow-up.

### 4.4. Mineral Metabolism Beyond iPTH

An important finding was the significantly lower calcium–phosphate product with etelcalcetide at 24 months (41.8 ± 6.8 vs. 53.2 ± 14.6; *p* = 0.023). However, this pattern, especially the significant calcium-lowering effect of etelcalcetide (serum Ca: 8.56 ± 0.47 vs. 9.25 ± 0.84 mg/dL at 24 months; *p* = 0.015) deserves careful interpretation. While lower calcium is a desired therapeutic effect in the context of CKD-MBD, it also carries the risk of hypocalcemia. In our study, this was managed by concurrent vitamin D supplementation and dialysate calcium adjustment, although despite this, 3 patients required dose adjustments and temporary breaks in treatment due to Calcium levels falling below 8.4. The Friedman test confirmed a significant time-dependent calcium decline with etelcalcetide (χ^2^ = 9.51, *p* = 0.009) but not with cinacalcet (χ^2^ = 1.56, *p* = 0.458), underscoring the more potent calcimimetic activity of etelcalcetide and the need for vigilant calcium monitoring during long-term therapy.

The significantly higher phosphate target attainment with etelcalcetide (85.7% vs. 28.6%; *p* = 0.004), while not a primary endpoint of this study, adds to the evidence that etelcalcetide may offer broader mineral metabolism correction beyond iPTH suppression alone [19]. These differences in mineral parameters were observed despite comparable rates of phosphate binder use (any calcium-containing binder: 61.9% vs. 66.7%; *p* = 1.000), dialysate calcium concentrations (1.50 mmol/L in 76.2% vs. 81.0%), and oral calcium supplementation (14.3% vs. 4.8%), suggesting that the observed effects are more likely attributable to the calcimimetic agents themselves than to differences in concomitant therapy.

### 4.5. Propensity Score Matching and Residual Confounding

A key methodological consideration in this study is the inherent confounding by indication in calcimimetic prescribing. Etelcalcetide, as a newer and intravenous agent, is preferentially prescribed for patients with more severe or cinacalcet-refractory SHPT; a phenomenon reflected in the higher baseline iPTH in the etelcalcetide group (1831 vs. 1377 pg/mL; *p* = 0.113) and the positive coefficient for baseline iPTH in the propensity score model (β = +0.566). Propensity score matching was employed to address this bias, achieving statistical balance across all individual covariates (all *p* > 0.10). However, some standardised mean differences exceeded the conventional ideal threshold of |SMD| < 0.10, most notably for baseline iPTH (SMD = +0.465) and hemodialysis duration (SMD = +0.394). Any residual confounding from higher baseline iPTH in the etelcalcetide group would be expected to bias results against etelcalcetide, as patients with more severe disease may be more difficult to treat. While this does not eliminate the possibility of unmeasured confounders operating in the opposite direction, the direction of the observed imbalance does not weaken the position of etelcalcetide.

### 4.6. Clinical Implications

The findings of this study have several practical implications for the management of SHPT in hemodialysis patients. First, the progressive widening of the efficacy gap between etelcalcetide and cinacalcet over time, from a medium to a medium-large effect at 24 months, suggests that short-term outcomes alone may not fully capture the relative effectiveness of these agents. Clinicians should consider the longitudinal trajectory of response, not just initial responder rates, when selecting a calcimimetic agent. Second, the lower inter-patient variability observed with etelcalcetide (CV ≈ 42% vs. ≈151%) may simplify clinical management if confirmed in larger studies. Third, the NNT of 2.3 for ≥50% iPTH reduction at 24 months indicates that nearly every other patient switched from cinacalcet to etelcalcetide would achieve a clinically meaningful additional response and a favourable therapeutic ratio in this patient population, though it requires confirmation in larger studies. Fourth, the composite mineral metabolism endpoint (50.0% vs. 14.3% achieving all three KDIGO targets) supports the concept of etelcalcetide as a more comprehensive CKD-MBD management tool, rather than solely an iPTH-lowering agent [10,11].

### 4.7. Limitations

This study has several important limitations that should be considered when interpreting the results. First, the retrospective, non-randomised design introduces the potential for unmeasured confounding. Although propensity score matching achieved adequate statistical balance (all *p* > 0.10), residual imbalances in baseline iPTH (SMD = +0.465) and hemodialysis duration (SMD = +0.394) could not be fully eliminated due to the limited matching pool.

Second, the sample size is modest (21 vs. 21 at 12 months, 14 vs. 14 at 24 months), which limits statistical power and the precision of effect estimates. Post-hoc power analysis indicated 55–58% power for the primary outcome, below the conventional 80% threshold. Non-significant *p*-values for some endpoints with medium-to-large effect sizes highlight the imprecision inherent in small-sample analyses. The effect estimates and their confidence intervals should be prioritised over *p*-value thresholds when interpreting these results. Future studies with larger cohorts are needed to confirm these findings with adequate power.

Third, while the multicentre design enhances external validity, it introduces the possibility of different clinical approaches, which cannot be accounted for. Fourth, no correction for multiple comparisons was applied across the secondary and exploratory endpoints. Consequently, the statistically significant findings among these analyses should be interpreted with caution and viewed as hypothesis-generating. Fifth, the significantly higher rate of dose modification in the etelcalcetide group (76.2% vs. 28.6%; *p* = 0.005) likely reflects the need for more frequent dose titration with intravenous calcimimetic therapy and complicates direct dose-equivalence comparisons. The numerically higher paricalcitol use in the etelcalcetide group (75.0% vs. 42.1%; *p* = 0.087) represents a potential confounder that could not be fully assessed.

### 4.8. Future Directions

The findings of this study provide a strong rationale for a multicentre, prospective, randomized controlled trial directly comparing etelcalcetide and cinacalcet over 24 months or longer in hemodialysis patients with SHPT. Additionally, pharmacoeconomic analyses comparing the total cost of care, including the elimination of oral medication costs, reduced clinic visits for adherence monitoring, and potential reductions in parathyroidectomy rates, would further inform clinical decision-making and reimbursement policies, particularly in middle-income countries such as Türkiye, where healthcare resource allocation is a significant consideration.

## 5. Conclusions

In this propensity-matched multicenter retrospective study, etelcalcetide was associated with a significantly greater and more sustained reduction in iPTH compared with cinacalcet over 24 months, along with lower inter-patient response variability. Sustained response (≥30% iPTH reduction at both 12 and 24 months) was nearly three-fold more frequent with etelcalcetide (78.6% vs. 28.6%; *p* = 0.021), and phosphate target attainment was significantly higher (85.7% vs. 28.6%; *p* = 0.004). However, these findings should be interpreted within the context of a modest sample size and retrospective design, and warrant confirmation in larger prospective trials.

## Figures and Tables

**Table 1:** Baseline characteristics of the cohort.

Variable	Etelcalcetide	Cinacalcet	*p*-Value
**12-month cohort**			
n	21	21	
Age (years)	48.6 ± 15.4	52.9 ± 11.9	0.320
Male sex, n (%)	13 (61.9)	11 (52.4)	0.756
HD duration (years)	9.4 ± 6.0	7.2 ± 4.8	0.246
iPTH (pg/mL)	1831.5 ± 1117.9	1377.0 ± 811.9	0.113
Ca (mg/dL)	9.09 ± 0.50	9.12 ± 0.61	0.840
P (mg/dL)	5.83 ± 1.14	5.80 ± 1.56	1.000
Ca × P product	53.2 ± 12.1	52.9 ± 15.2	0.980
**24-month cohort**			
Age (years)	47.5 ± 16.1	50.4 ± 12.8	0.730
Male sex, n (%)	8 (57.1)	8 (57.1)	1.000
HD duration (years)	8.5 ± 5.1	8.6 ± 4.8	0.963
iPTH (pg/mL)	1985.6 ± 1199.2	1619.4 ± 884.2	0.370
Ca (mg/dL)	9.18 ± 0.50	9.16 ± 0.67	0.963
P (mg/dL)	5.85 ± 1.22	6.08 ± 1.69	0.646
Ca × P product	54.0 ± 13.4	55.7 ± 16.9	0.730
**Concomitant therapy and calcimimetic dosing**			
	**intravenous, mg/week**	**oral, mg/day**	
Initial dose, median (range)	15.0 (7.5–30.0)	30 (30–60)	
Maintenance dose, median (range)	15.0 (2.5–30.0) †	30 mg/day: 15 (71.4%) 60 mg/day: 6 (28.6%)	
**Dose adjustments during follow-up**			
Dose increased, n (%)	6 (28.6)	5 (23.8)	
Dose reduced, n (%)	9 (42.9)	1 (4.8)	
Dose unchanged, n (%)	5 (23.8)	15 (71.4)	
Discontinued, n (%)	1 (4.8)	0 (0)	
Any dose modification, n (%)	16 (76.2)	6 (28.6)	0.005
Hypocalcemia-related pause, n (%)	6 (28.6)	—	
**Prior calcimimetic exposure (etelcalcetide group)**			
Prior cinacalcet use, n (%)	19 (90.5)	—	
Switched for intolerance, n (%)	3 (14.3)	—	
**Vitamin D analog therapy**			
Any vitamin D analog, n (%)	16 (76.2)	19 (90.5)	0.410
Paricalcitol	12 (57.1)	8 (38.1)	
Calcitriol	4 (19.0)	11 (52.4)	
None	5 (23.8)	2 (9.5)	
**Phosphate binder therapy**			
Non-Ca-based only, n (%)	7 (33.3)	7 (33.3)	
Ca-based only, n (%)	5 (23.8)	7 (33.3)	
Combination (Ca + non-Ca), n (%)	8 (38.1)	7 (33.3)	
None, n (%)	1 (4.8)	0 (0)	
Any Ca-containing, n (%)	13 (61.9)	14 (66.7)	1.000
**Dialysate calcium concentration**			
1.25 mmol/L, n (%)	1 (4.8)	2 (9.5)	
1.50 mmol/L, n (%)	16 (76.2)	17 (81.0)	
1.75 mmol/L, n (%)	4 (19.0)	2 (9.5)	
**Oral calcium supplementation**			
Oral calcium, n (%)	3 (14.3)	1 (4.8)	0.605

Data are presented as mean ± SD or n (%). *p*-values by Mann–Whitney U test (continuous) or Fisher’s exact test (categorical). † One patient discontinued etelcalcetide; maintenance dose reported for n = 20.

**Table 2:** Laboratory outcomes at 12 months (n = 21 per group).

Variable	Etelcalcetide (n = 21)	Cinacalcet (n = 21)	Diff [95% CI]	*p*-Value
**Within-group change from baseline (Wilcoxon signed-rank)**				
iPTH Δ (pg/mL)	−896.1	−466.2	—	<0.001/0.003
Ca Δ (mg/dL)	−0.01	−0.23	—	0.422/0.322
P Δ (mg/dL)	−0.43	−0.63	—	0.175/0.016
**Between-group at 12 months**				
iPTH (pg/mL)	935.4 ± 761.9	910.7 ± 786.9	24.6 [−458 to 508]	0.980
Ca (mg/dL)	9.09 ± 0.92	8.89 ± 1.07	0.20 [−0.4 to 0.8]	0.529
P (mg/dL)	5.40 ± 1.16	5.17 ± 1.26	0.23 [−0.5 to 1.0]	0.597
Ca × P	48.9 ± 10.4	45.8 ± 11.6	3.1 [−3.8 to 10.0]	0.352
**Change from baseline (between-group)**				
**%ΔPTH**	−50.5 ± 25.6	−29.7 ± 40.6	−20.8 [−42.0 to 0.5]	0.047 *
Cohen’s d	*−0.612 (medium)*			
**Absolute ΔPTH (pg/mL)**	−896.1 ± 721.9	−466.2 ± 757.4	−429.9 [−891 to 32]	0.007 **
Cohen’s d	*−0.581 (medium)*			
%ΔCa	+0.1 ± 11.0	−2.4 ± 11.0	2.5 [−4.4 to 9.4]	0.589
%ΔP	−4.9 ± 23.5	−7.1 ± 27.3	2.2 [−13.7 to 18.1]	0.450
**Responder rates—n/N (%) [ARD, 95% CI; OR, 95% CI]**				
≥30% iPTH reduction	14/21 (66.7%)	10/21 (47.6%)	19.0% [−10 to 48]	0.350
OR; NNT	2.20 [0.63–7.66]; 5.2			
≥50% iPTH reduction	11/21 (52.4%)	9/21 (42.9%)	9.5% [−21 to 40]	0.758
OR; NNT	1.47 [0.43–4.95]; 10.5			
KDIGO target (150–600)	11/21 (52.4%)	7/21 (33.3%)	19.0% [−10 to 48]	0.350
OR; NNT	2.20 [0.63–7.66]; 5.2			

Data are mean ± SD or n/N (%). Between-group *p*-values by Mann–Whitney U test (continuous) or Fisher’s exact test (categorical). Within-group *p*-values by Wilcoxon signed-rank test (Etel/Cin). 95% CIs for mean differences by Welch’s t-method; 95% CIs for ARD and OR by Wald method with Haldane correction. * *p* < 0.05; ** *p* < 0.01.

**Table 3:** Laboratory outcomes at 24 months (matched subset, n = 14 per group).

Variable	Etelcalcetide (n = 14)	Cinacalcet (n = 14)	Diff [95% CI]	*p*-Value
**Friedman test (0 → 12 → 24 months)**				
iPTH	χ^2^ = 22.29, *p* < 0.001	χ^2^ = 5.14, *p* = 0.076		
Ca	χ^2^ = 9.51, *p* = 0.009	χ^2^ = 1.56, *p* = 0.458		
P	χ^2^ = 6.65, *p* = 0.036	χ^2^ = 3.57, *p* = 0.168		
**Between-group at 24 months**				
iPTH (pg/mL)	787.7 ± 701.5	973.5 ± 830.3	−185.8 [−784 to 412]	0.535
Ca (mg/dL)	8.56 ± 0.47	9.25 ± 0.84	−0.69 [−1.2 to −0.2]	0.015
P (mg/dL)	4.88 ± 0.68	5.76 ± 1.52	−0.88 [−1.8 to 0.0]	0.069
Ca × P	41.8 ± 6.8	53.2 ± 14.6	−11.3 [−20.4 to −2.3]	0.023
**Change from baseline**				
%ΔPTH	−61.9 ± 26.0	−31.8 ± 47.9	−30.0 [−60.4 to 0.4]	0.057
Absolute ΔPTH (pg/mL)	−1197.9 ± 971.3	−645.9 ± 875.9	−552 [−1271 to 167]	0.085
%ΔCa	−6.4 ± 8.3	+1.6 ± 12.2	−8.0 [−16.1 to 0.2]	0.046
%ΔP	−13.8 ± 18.3	+0.2 ± 31.4	−14.1 [−34.3 to 6.1]	0.206
**Responder rates**				
≥30% iPTH reduction	11/14 (78.6%)	9/14 (64.3%)	14.3% [−19 to 47]	0.678
≥50% iPTH reduction	11/14 (78.6%)	5/14 (35.7%)	42.9% [10 to 76]	0.054
KDIGO target (150–600)	7/14 (50.0%)	4/14 (28.6%)	21.4% [−14 to 57]	0.440

Between-group comparisons by Mann–Whitney U test; responder rates by Fisher’s exact test. Friedman test assessed longitudinal change within each group.

## Data Availability

The data presented in this study are available on request from the corresponding author. The data are not publicly available due to patient privacy restrictions.

## Supplementary Materials

The following supporting information can be downloaded at https://www.mdpi.com/article/10.3390/jcm15176656/s1, Table S1: Propensity score matching details: logistic regression model, covariate balance before and after matching, and standardized mean differences; Table S2: Effect sizes, odds ratios, and numbers needed to treat; Table S3: Composite mineral metabolism endpoint at 24 months (matched subset, n = 14 per group).

## Abbreviations

The following abbreviations are used in this manuscript:

CKD	Chronic Kidney Disease
CKD-MBD	Chronic Kidney Disease–Mineral and Bone Disorder
SHPT	Secondary Hyperparathyroidism
RRT	Renal Replacement Therapy
iPTH	Intact Parathyroid Hormone
Ca	Calcium
P	Phosphate
Ca × P	Calcium–Phosphate Product
%ΔPTH	Percentage Change in iPTH from Baseline
KDIGO	Kidney Disease: Improving Global Outcomes
CaSR	Calcium-Sensing Receptor
HD	Hemodialysis
PSM	Propensity Score Matching
SMD	Standardized Mean Difference
CV	Coefficient of Variation
NNT	Number Needed to Treat
OR	Odds Ratio
CI	Confidence Interval
SD	Standard Deviation
IQR	Interquartile Range
SEM	Standard Error of the Mean
GBD	Global Burden of Disease
