# Supplementary material for: Etelcalcetide Versus Cinacalcet for Secondary Hyperparathyroidism in Hemodialysis Patients: A Multicentre Comparison with 24 Months of Follow-Up
Source: J Clin Med. 2026 Aug 28;15(17):6656. doi: 10.3390/jcm15176656 (PMC13566838; doi:10.3390/jcm15176656)
Supplement: Supplementary file 1 [file jcm-15-06656-s001.zip › jcm-4494482-supplementary.pdf]

Supplementary Materials

Etelcalcetide Versus Cinacalcet for Secondary Hyperparathyroidism in Hemodialysis Patients: A Multicentre Comparison With 24 Months of Follow-Up

Supplementary Table S1. Propensity score matching details: logistic regression model, covariate balance before and after matching, and standardized mean differences.

Panel A. Propensity Score Model (Logistic Regression)

Covariate	β (standardized)
Age	−0.337
Male sex	+0.405
HD duration (years)	+0.421
Baseline iPTH (pg/mL)	+0.566
Intercept	−0.028

PS, propensity score. Covariates were standardized (zero mean, unit variance) before model fitting. PS = predicted P(etelcalcetide | covariates).

Panel B. Propensity Score Distributions

	Etelcalcetide (n=21)	Cinacalcet (n=22)
Mean ± SD	0.561 ± 0.174	0.419 ± 0.163
Range	0.342 – 0.945	0.144 – 0.734
Overlap region	0.342 – 0.734	

Panel C. Covariate Balance — 12-Month Cohort (21 vs. 21)

Variable	Etel (n=21)	Cin (n=21)	SMD	p-value
Age (years)	48.6 ± 15.4	52.9 ± 11.9	−0.312	0.320
Male sex, n (%)	13 (61.9%)	11 (52.4%)	+0.189	0.756
HD duration (years)	9.4 ± 6.0	7.2 ± 4.8	+0.394	0.246
iPTH (pg/mL)	1831.5 ± 1117.9	1377.0 ± 811.9	+0.465	0.113
Ca (mg/dL)	9.09 ± 0.50	9.12 ± 0.61	−0.044	0.840
P (mg/dL)	5.83 ± 1.14	5.80 ± 1.56	+0.027	1.000
Ca×P	53.2 ± 12.1	52.9 ± 15.2	+0.027	0.980
Propensity score	0.561 ± 0.174	0.425 ± 0.164	+0.776	0.029

SMD, standardized mean difference. Balance: Good (|SMD| < 0.10), Adequate (|SMD| 0.10–0.50), Poor (|SMD| > 0.50). All individual covariates achieved p > 0.10. † Residual PS imbalance reflects the baseline iPTH difference — a consequence of confounding by indication, as etelcalcetide is preferentially prescribed for more severe SHPT. See Limitations.

Panel D. Covariate Balance — 24-Month Matched Subset (14 vs. 14)

Variable	Etel (n=14)	Cin (n=14)	SMD	p-value
Age (years)	47.5 ± 16.1	50.4 ± 12.8	−0.183	0.730
Male sex, n (%)	8 (57.1%)	8 (57.1%)	0.000	1.000
HD duration (years)	8.5 ± 5.1	8.6 ± 4.8	+0.072	0.963
iPTH (pg/mL)	1985.6 ± 1199.2	1619.4 ± 884.2	+0.352	0.370
Ca (mg/dL)	9.18 ± 0.50	9.16 ± 0.67	−0.014	0.963
P (mg/dL)	5.85 ± 1.22	6.08 ± 1.69	−0.080	0.646
Ca×P	54.0 ± 13.4	55.7 ± 16.9	−0.056	0.730
Propensity score	0.578 ± 0.178	0.465 ± 0.155	+0.313	0.629

All individual covariates achieved $p > 0.10$ and $|SMD| < 0.40$. The 24-month subset shows improved balance compared to the 12-month cohort due to the availability of a larger matching pool (21 → 14).

Supplementary Table S2. Effect sizes, odds ratios, and numbers needed to treat.

Measure	Value
Cohen's d (negative favors etelcalcetide)	
%ΔPTH at 12 months (21 vs. 21)	d = −0.612 (medium)
%ΔPTH at 24 months (14 vs. 14)	d = −0.779 (medium–large)
Absolute ΔPTH at 12 months	d = −0.581 (medium)
Absolute ΔPTH at 24 months	d = −0.597 (medium)
Odds ratios (etelcalcetide vs. cinacalcet)	
≥50% iPTH reduction at 12 months	OR = 1.47 (95% CI: 0.43–4.95)
≥50% iPTH reduction at 24 months	**OR = 6.60 (95% CI: 1.23–35.44)**
KDIGO target at 12 months	OR = 2.20 (95% CI: 0.63–7.66)
KDIGO target at 24 months	OR = 2.50 (95% CI: 0.52–11.93)
Number needed to treat	
≥30% reduction at 12 months	ARD = 19.0%, NNT = 5.2
≥50% reduction at 12 months	ARD = 9.5%, NNT = 10.5
≥30% reduction at 24 months	ARD = 14.3%, NNT = 7.0
≥50% reduction at 24 months	**ARD = 42.9%, NNT = 2.3**

ARD, absolute risk difference; NNT, number needed to treat; OR, odds ratio; CI, confidence interval.

Supplementary Table S3. Composite mineral metabolism endpoint at 24 months (matched subset, n = 14 per group).

**Component**	**Etelcalcetide**	**Cinacalcet**	**p-value**
iPTH 150–600 pg/mL	7/14 (50.0%)	4/14 (28.6%)	0.440
Ca 8.4–10.2 mg/dL	12/14 (85.7%)	10/14 (71.4%)	0.640
P 3.5–5.5 mg/dL	12/14 (85.7%)	4/14 (28.6%)	0.004
**All three met (composite)**	7/14 (50.0%)	2/14 (14.3%)	0.103
Composite OR (95% CI)	6.00 (0.97–37.30)		

Fisher's exact test for individual components and composite; Wald method for OR.
